# Confessions and Denials When Guilty and Innocent: Forensic Patients' Self-Reported Behavior During Police Interviews

**DOI:** 10.3389/fpsyt.2019.00168

**Published:** 2019-03-29

**Authors:** Renate Volbert, Lennart May, Joscha Hausam, Steffen Lau

**Affiliations:** ^1^Charité-–Universitätsmedizin Berlin, Institute of Forensic Psychiatry, Berlin, Germany; ^2^Psychologische Hochschule Berlin, Berlin, Germany; ^3^Department of Forensic Psychiatry, University Hospital of Psychiatry, University of Zurich, Rheinau, Switzerland

**Keywords:** police interview, suspect, interrogation, false confessions, forensic patients, denial, self-report, mental illness

## Abstract

Several self-report studies together with analyses of exoneration cases suggest that suspects with mental disorder are especially prone to making false confessions. The present study asked 153 forensic patients in Germany about their behavior during suspect interviewing by the police. Self-reported ground truth of guilt and innocence was asked for, thereby taking into account that the risk of false confession is present only if a person has ever been interviewed when innocent. Indeed, surveying samples that include suspects who have never been interviewed when innocent may lead to underestimating the risk of false confessions. In the present study, all patients reported having been interviewed previously when guilty; and almost two-thirds (62%, *n* = 95), that they had also been interviewed at least once when innocent. These participants stated that they remained silent while being interviewed significantly more often when guilty (44%) compared to when innocent (15%). This corroborates laboratory research findings indicating that the right to remain silent is waived more often by innocent than by guilty suspects. Out of all 95 participants who were ever interviewed when innocent, 25% reported having made a false confession on at least one occasion. This result is in line with previous international research showing a high percentage of false confessions among suspects with mental disorder.

## Introduction

The fact that suspects really do make false confessions has been confirmed repeatedly in recent years [e.g., (1, 2)]. However, for many reasons, it is difficult, if not impossible, to ascertain what the objective rates of false confessions might be, which offenses are confessed to falsely, and how frequently which different causal backgrounds emerge. Although most available knowledge on false confessions comes from analyses of exoneration cases (3, 4), self-report studies offer a further methodological approach.

### Prevalence Estimations of False Confessions Based on Analyses of Exoneration Cases

Founded in 2012, the National Registry of Exonerations has been documenting information about exonerations occurring in the United States from 1989 to the present (by 26.01.2019, this had amounted to 2,364 cases; www.law.umich.edu/special/exoneration/Pages/about.aspx). For all offenses, the Registry reveals an average of 12% false confessions. At 23%, the proportion of false confessions for homicide is higher than that for all other offenses. However, a clear external criterion for the actual innocence of the exonerees does not exist for all cases listed in the National Registry of Exonerations. The Innocence Project in contrast, registers only cases in which the innocence of a previously convicted person has been confirmed by DNA analysis (362 cases by 26.01.2019; http://www.innocenceproject.org). Evaluation of these cases, which are primarily violent crimes and sex offenses, reveals that in 28% of DNA exonerations, false confessions were a contributing factor (https://www.innocenceproject.org/dna-exonerations-in-the-united-states/).

In Germany, Peters (5, 6) analyzed about 963 cases of retrials between the years 1951 and 1964. In 724 of these cases, retrial led to an acquittal. Peters found that around 7% of the defendants who were acquitted in the retrial had originally made a confession. Assuming that the subsequently acquitted individuals actually were innocent, these are false confessions. However, as in the National Registry of Exonerations, no external criterion of actual innocence is available for all members of this German sample. Nonetheless, analyzing only a subsample that is more comparable to the sample in the Innocence Project reveals a similar proportion to that found in the United States: within the German sample, a false confession could be found in 5 out of 21 homicide cases (24%) in which the convicted was exonerated in the retrial proceeding after his or her innocence was confirmed. However, this investigation is more than 40 years old, and no recent information is available on the frequency of false confessions in Germany.

Analyses of exoneration samples refer typically to (a) cases of serious crimes that have a low base rate and (b) police-induced false confessions (7) that were mostly withdrawn already at the end of the police interview but nonetheless led to a—false—conviction (3). In contrast, voluntary false confessions, in which somebody confesses in the absence of any interrogation influence, are hardly ever found in exoneration samples; they may either not be prosecuted or convicted in the first place, or they are not withdrawn and no retrial is sought.

### Self-Reported Prevalence of False Confessions

Several studies have gathered self-reported information on false confessions in community samples as well as in samples of prison inmates. These reveal some consistent trends in the self-reported prevalence of false confessions [see, for a summary, (8, 9)]:

- Apart from one study in which nobody reported ever making a false confession (10), all studies—including those in community samples—revealed a small, but in no way negligible percentage of respondents who reported having already made at least one false confession (between 1.2 and 13.8% in community samples).
- When persons were surveyed who had already been interviewed repeatedly as suspects by the police, the proportion reporting having made a false confession in the past was larger and was almost always more than 10% and sometimes even more than 20% [between 6 and 24%; (8)].

However, several of the self-report studies were carried out in Iceland. Because false confession rates probably depend strongly on the given national boundary conditions (e.g., police interviewing practices), it is questionable whether these findings generalize to other countries. Although surveys from other countries are available (Bulgaria, Finland, Latvia, Lithuania, Norway, and Russia; 9), that also indicate differences between countries, findings are generally similar. However, no studies on self-reported false confession rates are available for Germany.

The most commonly *self-reported motives* for false confessions are succumbing to police pressure, protecting another person (the actual offender), and avoiding police detention (11). False confessions are self-reported most frequently for property offenses and serious traffic violations (11). In a study with mentally ill offenders (12), participants claimed that they made false confessions in order to stop police questioning, to protect the true perpetrator, because they succumbed to police pressure, or because they initially believed they were involved.

For logical reasons, suspects face the risk of making a coerced false confession only if the following preconditions are given: (a) the police suspects and interviews an innocent person; (b) the suspect waives the right to remain silent; and (c) there might be incriminating evidence, but evidence that *definitely proves* the guilt of the suspect is missing and cannot exist because of the suspect's innocence (13). Existing studies often neglect the very trivial fact that the risk of a false confession emerges only if a person has ever been interviewed when innocent. Hence, surveys of samples that include suspects who have never been interviewed when innocent may well-underestimate the prevalence of false confessions.

### Risk Factors for False Confessions

Studies on factors that may increase the likelihood of a false confession refer mostly to confessions that are policed-induced. These focus on *investigative risk factors* and refer to the police investigators' cognitive processes and behavior along with the influence of specific measures such as custody. Reviews of this literature can be found in Gudjonsson (14), Kassin (15), and Kassin et al. (2).

There are also *personal risk factors* for false confessions. These are personal characteristics of the suspect such as, in particular, young age, and mental disorder. Gross and Shaffer (4) analyzed 873 cases registered in the National Registry of Exonerations and found that youths and persons with mental impairment were particularly vulnerable: whereas the false confession rate among adults with no known psychological disorders or mental impairment was 8% (56/719); that among the under-18s, was 42% (39/92); and that among persons with a psychological disorder or mental impairment, was even 75% (53/70).

Mental problems have also been identified as a risk factor in self-report studies. Two out of three studies in which more than 20% reported having made a false confession in the past have investigated persons with mental disorders [(12): 22%; (16) cited in (8): 23%]. Participants claiming to have confessed falsely also reported having higher levels of illicit drug use and substance misuse treatment; more adverse life events (17); and higher levels of victimization, anxiety, depression, and anger (18) compared to people who never made a false confession. Self-reported false confessors were also found to have more antisocial personality characteristics than non-false confessors (10).

Self-report studies also show that false confessions occur more often among persons with a delinquent lifestyle: in comparison to non-false confessors, false confessors had been interviewed more often as suspects (19), arrested more often (11), sentenced more often to life imprisonment, had more years of offending (12), served more frequently and longer in prison, were younger at the time of their first criminal conviction and imprisonment (20), and had more delinquent peers (18). However, these variables might reflect at least in part the fact that people with a more extensive criminal history are interviewed more often by the police, and this, in turn, may increase the probability that they will be suspected falsely.

### True Confessions

To identify specific features of false confessions, they have to be compared with the conditions in which true confessions emerge. Unfortunately, there are still surprisingly few estimates of how often suspects confess at all during police interviewing. Moston and Emgelberg (21) found that general confession rates ranged from 42 to 64% in the United States and from 55 to 62% in Great Britain.

Nonetheless, confession rates vary greatly depending on the sample. In Germany, Bippert (22) analyzed the files of 106 male defendants who had been convicted of homicide offenses, and found that 67% confessed during the course of the police interview. In retrospective interviews with 56 incarcerated male adolescents, Kraheck-Brägelmann (23) found that 45% made a full and 50% a partial confession. In a self-report study, Sigurdsson and Gudjonsson (24) also found a true confession rate of 92% among Icelandic prison inmates. However, these studies analyzed only cases that were prosecuted further. Thus, they probably included a disproportionately high number of cases in which strong evidence might have affected the decision to confess.

A review of all 743 suspects (irrespective of whether or not cases were further prosecuted) from one police department in Germany across the span of 1 year—thus including a variety of offenses—showed that 35% confessed, 15% denied the alleged offense, and 50% made no statement (25). A study in Great Britain with unselected police interviews revealed a confession rate of 39% (26). Note that these studies were unable to control whether either allegations or confessions were true or false.

Reported reasons for true confessions are, among others, the perceived strength of evidence (21, 27, 28), a need to clear one's conscience, police pressure (24), custodial pressure, and a desire to be released from police detention (29).

Research contrasting true and false confessions is almost non-existent. One exception is Sigurdsson and Gudjonsson's (10) within-sample analysis of 51 alleged false confessor inmates in Iceland. This compared the false confession experience with the experience of their current true confession. Significant differences were found for external pressure (found to be higher for false than for true confessions), internal pressure, perception of proof, and legal rights (all found to be higher for true confessions). Another exception is Redlich et al. (30) between-subject design with which they studied 30 true and 35 self-reported false confessors with mental illness in the United States. False confessors reported significantly more external and less internal pressure than true confessors. Taken together, true confessions show at least a partial overlap with the factors associated with false confessions (14). However, true and false confessions differ in terms of the prevalence and distribution of the reported motives.

The current study is the first to survey the behavior of forensic patients in suspect police interviews in Germany. Forensic patients are offenders who are ordered by criminal courts to either (a) detention and treatment in a secure psychiatric hospital (section 63 of German Criminal Law) or (b) detention in a secure custodial addiction treatment unit (section 64 of German Criminal Law). As pointed out above, the available studies suggest that false confessions are particularly frequent in this population. The goal of the present study was to determine whether a German sample would reveal a comparable proportion of false confessions to that found within this group in international studies. In one aspect, the present study goes beyond most previous research on self-reported false confessions: it takes into account that a risk of making a police-induced false confession is given only if suspects are interviewed when innocent and waive their right to remain silent. Therefore, we asked participants explicitly whether they had ever been interviewed by the police when they were innocent. Subsequently, we analyzed self-reported participants' behavior separately if interviewed when guilty vs. if interviewed when innocent; and we also calculated the proportion of truthful (true confessions and true denials) and false statements (false confessions and false denials) as well as the proportion of patients exercising their right to remain silent. We consider that viewing the proportion of self-reported false confessions as the proportion of cases in which someone has been interviewed when innocent provides a more appropriate estimate of the risk of making a false confession than estimates based on samples that include participants who have never been interviewed when innocent. By asking participants about their behavior while being interviewed when guilty and being interviewed when innocent, we also gathered information on true confessions, false denials, or decisions to remain silent.

## Methods and Materials

### Participants

Participants were 153 patients (7 female and 146 male) with a mean age of 33.69 years (*SD* = 10.71, range from 20 to 67) detained in six forensic hospitals in Germany. Originally, 159 patients were recruited, but 6 were dropped because of language/communication difficulties. Patients had been convicted for violent offenses (82%), sexual offenses (24%), property offenses (27%), drug offenses (10%), and other offenses not covered by these categories (27%; multiple offenses possible). The distribution of ICD diagnoses was as follows (multiple diagnoses possible): mental/behavioral disorders due to psychoactive substance use (68%); personality disorders (31%); mental retardation (22%); schizophrenia, schizotypal, and delusional disorders (12%); and others (5%).

### Procedure

The study was approved by the Ministries of Social Affairs and Health responsible for forensic hospitals in the respective Federal States. Patients were informed about the survey by their physician or psychotherapist. Those willing to participate were asked to register on a list. Participants were interviewed individually by one of four interviewers. Before the beginning, interviewers explained the purpose of the study again, assured patients that their data would be used anonymously, and emphasized that they could drop out of the study at any time without having to fear any negative consequences. After that, patients were asked whether they were willing to sign a consent for both the interview and access to information on their current diagnosis (ICD-10) and sentence. After they consented, the questionnaire was read aloud and patients' answers were documented. Patients whose capability to give informed consent was questionable were not interviewed. Interviews lasted between 5 and 30 min depending on how many police interview constellations were reported by the patient.

### Measures

#### Questionnaire

A questionnaire was developed to gather information on the patients' behavior during police interviews. The first question asked whether the police had ever interviewed them as a suspect for an offense they had actually committed. If they answered in the affirmative, participants were asked how often they were interviewed in this way and whether they had remained silent, denied the offense, or confessed during these suspect interviews. Those who claimed to have confessed were asked about the reasons for their true confessions (presented in a yes/no format; multiple reasons were possible, see Appendix).

Next, patients were asked whether the police had ever interviewed them as a suspect for an offense they had not committed. If they answered in the affirmative, they were asked how often this had occurred and whether they had remained silent, denied the offense, or confessed during the interview. If suspects claimed they had made a false confession, they were asked about the number of false confessions, the offenses, and the reasons for these false confessions (yes/no format; multiple answers possible, see Appendix). Furthermore, they were asked about their age at the time of the false confession. We also asked patients whether they had been in a prison or in a forensic hospital before.

When answering questions on the reasons for making true or false confessions, patients were given a list of categories derived from the literature [see also (12)]. These categories could be subdivided further: suspects might, for example, infer mitigation because of wishful thinking, because minimization tactics were used, or because an explicit promise was made. However, given that these are retrospective data gathered from a relatively small sample of people with mental health problems, such distinctions were not made. Because the available studies indicate that reasons for making true or false confessions overlap, and the current study aimed to compare reasons for making true confessions with those for making false confessions, set response categories were preferred to an open response format.

#### Information on Current Sentence and Diagnoses

Information on patients' current sentences and diagnoses (ICD-10) was provided by the responsible psychiatrist.

## Results

All participants confirmed that the police had interviewed them over at least one offense they had actually committed, with a range from 1 to 150 interviews (*M* = 14.71, *SD* = 23.14, *Mdn* = 6.00). A total of 95 (62%) reported that they were also interviewed as a suspect over at least one offense they had not committed, with a range from 1 to 50 interviews (*M* = 3.04, *SD* = 5.65, *Mdn* = 2.00).

Patients who reported having been interviewed when both guilty and when innocent did not differ significantly in terms of prior imprisonment, prior forensic treatment, or diagnoses from those who stated that they had never been interviewed when innocent—with one exception: the percentage of patients diagnosed with schizophrenia, schizotypal, and delusional disorders was significantly smaller in the subsample with guilty and innocent interviews (6%) than in the subsample with only guilty interviews (20%; Fisher exact test *p* = 0.015).

### Self-Reported Confessions, Denials, and Exercising the Right to Remain Silent

Table 1 displays the distribution of confessions, denials, and exercises of the right to remain silent in both conditions. Because measurements were repeated in the subsample of 95 patients who were interviewed when both innocent and guilty, whereas this was not the case for the remaining 58 patients, individual McNemar's chi-square tests were computed for each of the three interview variants (confessions, denials, exercise of the right to remain silent) in the 95 patients who reported both types of interviews (guilty/innocent). Results showed significant differences (all *p* < 0.001) between interviews in which patients were interviewed when guilty vs. innocent for all three interview variants. Participants reported more confessions when guilty (78%) than when innocent (25%), and more denials when innocent (79%) than when guilty (35%). They also reported significantly more frequently waiving their right to remain silent when innocent (85%) than when guilty (56%).

**Table 1:** Reported behavior during interviews when guilty and when innocent (in brackets: frequencies for the subgroup that reported having been interviewed when both guilty and innocent).

	**Interviewed when guilty (*N* = 153) [*n* = 95]**	**Interviewed when innocent (*n* = 95)**
Remained silent	57 (37%) [42 (44%)]	14 (15%)
Denied	47 (31%) [33 (35%)]	75 (79%)
Confessed	124 (81%) [74 (78%)]	24 (25%)

*Multiple answers possible; therefore, percentages do not add up to 100. Number of affirmative answers (n)*.

### False Confessions

A total of 24 patients reported having made at least one false confession. This represents 16% of the whole sample (24/153). But this percentage is somewhat misleading, because 58 patients were not at risk of confessing falsely, having never been interviewed when innocent. How these 58 patients would have behaved had they been interviewed when innocent remains an open question. Of the 95 participants who had been interviewed when innocent, 25% (24/95) reported having confessed falsely at least once. The prevalence rate increased further to 28% when including only those participants who (a) were interviewed when innocent and (b) made a statement (24/86). These 24 patients reported a total of 38 false confessions, ranging from 1 to 7 false confessions per patient. Fifteen of the 24 patients reported having been convicted after making a false confession. False confessions referred to property offenses (*n* = 15), violent offenses (*n* = 7), homicide (*n* = 2), drug offenses (*n* = 2), and other offenses not covered by these categories (*n* = 7).

### Reasons for Confessions

“Strong evidence” (65%) and “hope for mitigation of sentence” (41%) were the most frequently reported reasons for *true* confessions, whereas the most frequent reason for *false* confessions was “protecting the real perpetrator” (63%). A substantial minority of participants reported “hope for release from custody,” “interviewing pressure,” and “feeling of physical discomfort (e.g., being overtired)” as reasons for true and for false confessions (Table 2). The group of 24 false confessors was, in a sense, divided: the 15 patients who claimed to have protected the actual perpetrator named only three other reasons (2 x being pressured by the real perpetrator, 1 x hope for mitigation of sentence). All other reasons were given by the remaining 9 false confessors.

**Table 2:** Self-reported reasons for true and false confessions.

	**True confessions (*n* = 124)**	**False confessions (*n* = 24)**
Evidence was strong^a^/Police claimed evidence to be strong^b^	80 (65%)	4 (17%)
Hoped for mitigation of sentence	51 (41%)	4 (17%)
Wanted to ease conscience^a^/Feeling of guilt^b^	35 (28%)	0
Hoped to be released from custody	24 (19%)	3 (13%)
Because of the interviewing pressure	16 (13%)	6 (25%)
Did not feel well physically, e.g., overtired	14 (11%)	2 (8%)
Protecting the real perpetrator	(not asked)	15 (63%)
Being pressured by the real perpetrator	(not asked)	2 (8%)
To gain attention	(not asked)	1 (4%)
Was convinced to be the perpetrator	(not asked)	0
Others	28 (23%)^c^	5 (21%)^d^

a *Asked for true confessions only*.

b *Asked for false confessions only*.

c For example: “I am not able to lie” or “My family should not know.”

d For example: “I did it out of love” or “I wanted to be part of the clique.”

*Multiple answers possible; therefore, percentages do not add to 100*.

### Differences Between Confessors and Non-confessors

Fisher exact tests were calculated to examine differences in diagnoses and prior delinquency between (a) guilty confessors and guilty non-confessors and (b) innocent false confessors and innocent non-confessors. Results are displayed in Table 3.

**Table 3:** Frequencies and percentages of ICD-10 diagnoses and self-reported delinquency by confessors and non-confessors split for interviews when guilty and innocent.

	**Interviewed when guilty (*****n*** **=** **153)**		**Interviewed when innocent (*****n*** **=** **95)**	
	**Confessed (*n* = 124)**	**Not confessed (*n* = 29)**	**Confessed (*n* = 24)**	**Not confessed (*n* = 71)**
	***n* (%)**	***n* (%)**	***n* (%)**	***n* (%)**
**MENTAL DISORDERS (ICD-10)**				
F10–F19 (due to substance use)	79 (64%)	25 (86%)^*^	17 (71%)	48 (68%)
F20–F29 (Schizophrenia)	14 (11%)	4 (14%)	2 (8%)	4 (6%)
F60–F69 (Personality disorders)	40 (32%)	8 (28%)	8 (33%)	22 (31%)
F70–F79 (Mental retardation	29 (23%)	4 (14%)	4 (17%)	17 (24%)
Others	11 (8%)	1 (3%)	1 (4%)	7 (10%)
**PRIOR DELINQUENCY**				
Prior imprisonment	70 (56%)	20 (69%)	17 (71%)	40 (57%)
Prior forensic treatment	21 (17%)	4 (14%)	5 (21%)	14 (20%)

* *p < 0.05*.

#### Guilty Confessors vs. Guilty Non-confessors

Compared to guilty confessors, guilty non-confessors were more often diagnosed with a mental and behavioral disorder due to psychoactive substance use (ICD-10 F10–F19; 64 vs. 86%; Fisher exact test, *p* = 0.026). No other significant differences were found.

#### Innocent False Confessors vs. Innocent Non-confessors

No significant differences were found.

## Discussion

The current study examined German forensic patients' self-reports on their behavior while being interviewed by the police when either innocent or guilty. All patients stated that they were interviewed by the police for at least one offense they had actually committed. Almost two-thirds of the forensic patients (62%) reported that they had also been interviewed at least once in the past when innocent. Little is known about how often people undergo a suspect interview when they are actually innocent. The results of our study suggest that being interviewed as a suspect when innocent is not a rare experience for people with a criminal record.

Most patients in the current study (81%) reported having made a true confession when they were guilty during at least one of the police interviews. The majority also reported having made a truthful statement during police interviews when innocent (79% true denials). Taken together, the most frequent behavior during police interviews was reported to be making a truthful statement, irrespective of whether patients were guilty or innocent [see also (9)]. Notwithstanding, a substantial proportion of false denials (31%) and false confessions (25%) was also revealed.

### True Confessions

The prevalence of true confessions (81%) in the current study exceeded the range of confession rates found in international studies [43–76%; (21)]. However, studies with samples of incarcerated inmates have also revealed comparable or even higher prior confession rates (20, 23).

### False Confessions

The percentage of self-reported false confessions within the whole sample was 16% (24/153) and thus in the range of comparable surveys of prison inmates in other countries [6–24%: (8)]. Nonetheless, the proportion of false confessions within the whole sample was slightly lower than the 22% reported by Redlich et al. (12) who also surveyed a sample of offenders with mental disorders.

However, by calculating a proportion of self-reported confessions within a whole sample, one might well-underestimate the actual prevalence: suspects face the risk of making a false confession only if they are (a) interviewed when innocent and (b) waive their right to remain silent. This is at least true for police-induced confessions. People can, however, come forward to the police with a voluntary false confession without being suspected before. In these cases, a suspect interview would not be conducted without the confession in the first place. From the examples patients mentioned during the survey, it can nonetheless be assumed that this was often not the case in the current sample. Participants reported on cases in which, for example, they knew the real perpetrator but were mistaken for the culprit by the police. Or they were interviewed for an offense they actually had committed while they were additionally alleged to have committed a crime for which they were not responsible.

Whereas, the percentage of self-reported false confessions within the whole sample was 16%, this rose to 25% (24/95) in the subgroup of participants who had at some time been interviewed when innocent. Put differently, one in four forensic patients who ever had the opportunity to make a false confession claimed to have done so. Out of those suspects who made a statement while being interviewed when innocent, 28% (24/86) reported having falsely confessed in at least one of these interviews. Altogether, we view the proportion of false confessions that refer solely to interviews when innocent as a more appropriate estimate for the risk of making a false confession in a police interview than the proportion reported in most previous studies that gave only the overall prevalence (proportion of false confessions within the whole sample).

In line with other self-report studies, property offenses were the most common type of offenses for which the patients claimed to have confessed falsely [e.g., (11)].

### Waiving the Right to Remain Silent

Whether patients denied, confessed, or exercised their right to remain silent during the police interviews differed significantly between interviews when guilty and when innocent. For denials and confessions, these results are rather trivial. Less trivial, however, is the result that significantly more patients stated that they had waived their right to remain silent at least once while being interviewed when innocent (85%) compared to interviewed when guilty (63% when looking at the total sample; 56%, when looking at the 95 participants who were interviewed when both guilty and innocent). This result is in line with Kassin's claim that “innocence puts innocents at risk” [2005; see also (2)]. A series of experimental studies have demonstrated that innocent suspects are more forthcoming than guilty suspects [e.g., (31, 32)]. Hence, the field data from the current study support these previous findings.

### Reasons for Confessions

Focusing on the suspects' confessions, we asked them why they gave a true or false confession. Overall, the most frequently reported reason for a *false confession* was to protect the real perpetrator (*15/24;* 63%). This reason for false confessions is frequently reported in all self-report studies. However, the percentage in the current study was even higher than in other self-report studies (11, 12). In contrast, interviewing pressure (6/24; 25%) was claimed less frequently in comparison to the percentages reported in international studies [e.g., (11, 12)]. Whether these differences are due to differences in the way police carry out their interviews in different countries or to different samples characteristics cannot be determined from the current data.

It should, however, be emphasized that police interviewing pressure still constitutes the second most frequently reported reason for a false confession in the current study. Moreover, a combination of different situational factors resulting from police interrogation tactics (interviewing pressure, claims by the police that the evidence was strong, hope to be released from custody, hope for mitigation of sentence) were reported by almost 40% of all cases (9/24). These factors point to substantial social-psychological influences. Even when protecting the real perpetrator is given as the reason, in many cases such constellations do not represent the classic version of a voluntary false confession in which a person confesses in the absence of any external influence (7). It is far more often the case that these patients were interviewed as suspects for an offense that was actually committed by a person they knew and that they then confessed during the police interview. Many of these patients would possibly not have falsely confessed without the situational effects of interrogation.

With respect to *true confessions*, the most commonly reported reason was that the evidence was strong. This suggests that strength of evidence is crucial for the decision to make a true confession, and this is once more in line with existing empirical evidence (21).

Taken together, some motives were inherently found to be exclusive to true confessions (evidence was actually strong) or false confessions (protecting the real offender, being pressured by the real offender), but there is also an overlap of motives reported for both kinds of confession (interview pressure by the police, hope for release from custody). Nonetheless, possible responses were limited by the categories used in this study. To explore the reasons for true and false confessions in a more differentiated way, future research should include interviews with true and false confessors.

### Differences Between Confessors and Non-confessors

In the current study, prior imprisonment tended to be more prevalent among innocent false confessors compared to innocent non-confessors (71 vs. 57%). However, this difference was not statistically significant. Although this lack of significance may be due to the small number of cases, one might also argue that a longer criminal history will be associated with a higher number of suspect interviews and thus with a higher probability of being interviewed when innocent. Previous findings showing an association between criminal history and false confession (11) might simply reflect the heightened probability of being interviewed occasionally when innocent. However, in the current sample, participants who were interviewed only when guilty did not differ in terms of prior imprisonment and prior forensic treatment from those who were interviewed when guilty and when innocent.

Patients diagnosed with a mental retardation did not self-report higher false confession rates than other patients. This was rather unexpected in light of existing research on the vulnerability of suspects with low intelligence [e.g., (33)]. The present result may be due partly to a self-selection process: patients with more severe intellectual deficits probably did not volunteer to participate in this study or could not be included because they lacked the capacity to give informed consent or had difficulties in understanding and answering the questions.

### Limitations

Some limitations have to be addressed: first, the sample probably does not represent the population of forensic patients in Germany. Participation required sufficient intellectual capacity to understand the questionnaire, maintain attention for a period of time, and communicate with basic German language skills. This might have excluded patients who may be even more prone to false confessions. Second, reports are based on persons and not interviews. Asking participants to report an interview behavior that they had displayed at least once in the past may have distorted the data on participants with multiple police interviews. They may well have shown the reported behavior only in one exceptional situation that deviated from their typical—more frequently shown—interview behavior. However, this approach can certainly be used as a basis to estimate whether a certain interview behavior (e.g., a false confession) has *ever* been shown. Nonetheless, the study is further limited by the questionable validity of self-report information with its susceptibility to motivational and memory errors. There is a lack of external criteria to corroborate the reported information on behavior during police interviews. Nevertheless, it should be emphasized that the percentages of self-reported false confessions in international studies are quite similar to those in the current study. In addition, those groups that have already proven to be vulnerable on the basis of exoneration file studies also prove to have higher false confession rates in self-report studies than others, and this can be viewed as supporting the validity of the self-report data.

## Conclusions

Results of the current study confirm previous international self-report studies showing that a false confession is not a rare event. Rates are similar to those found in the international literature on persons with mental disorder.

Until recently, these findings had received little attention in German law enforcement practice (34). However, in 2017, the German Parliament passed legislation requiring certain suspect interviews by the police, including interviews of underage suspects and of suspects with mental disorder or disorders, to be audio- or videotaped from 2020 onward (35).

The current study shows—together with other self-report studies [e.g., (12)]—that examining only exoneration cases might lead to the wrong impression that false confessions occur mainly in response to severe allegations. Proven false confessions in exoneration cases typically refer to offenses such as homicide or sexual assault that have a low base rate. In contrast, self-report studies suggest that false confessions occur frequently in more prevalent but less serious crimes. Self-reported false confessors name the protection of the true perpetrator as a frequent reason for a false confession [see also (8, 9)]. However, this does not necessarily mean that people enter the police station and confess to a crime despite never being suspected. In contrast, the patients were often erroneously interviewed as suspects for an offense that was actually committed by a person they knew, and they only made a false confession during the course of the police interview. Because many of the false confessors also stated reasons that indicate the situational effects of the suspect interviews, substantial social-psychological influences should be assumed in these cases. Based on the current data, it is not possible to state conclusively whether the circumstance that the patients knew the true offender was the actual reason for a false confession; or made them more vulnerable to interrogative influences in the sense that a false confession may constitute the alternative to betraying a peer. Future research should address this question by including interviews with innocent confessors and non-confessors.

## Data Availability

The raw data supporting the conclusions of this manuscript will be made available by the authors, without undue reservation, to any qualified researcher.

## Author Contributions

RV and SL contributed the conception and design of the study. LM, JH, and SL organized the data acquisition and carried out data collection. LM performed the statistical analysis. LM wrote the first draft of the manuscript. RV wrote parts of the manuscript and revised the draft. All authors read and approved the submitted version.

### Conflict of Interest Statement

The authors declare that the research was conducted in the absence of any commercial or financial relationships that could be construed as a potential conflict of interest.

## Appendix

Why did you truly confess?

Because the evidence was strong.

I wanted to ease my conscience.

Because of the interviewing pressure.

I was hoping to get released from custody.

I was hoping to get a more lenient punishment.

I was physically not well (e.g., overtired)

Other:

Why did you falsely confess?

I wanted to protect the real perpetrator.

I was pressured by the real perpetrator to confess.

Because of the interviewing pressure.

The police claimed the evidence to be strong.

I was physically not well (e.g., overtired)

I was hoping to get released from custody.

I was hoping to get a more lenient punishment.

I wanted to attract attention.

I felt guilty.

I was convinced to be the perpetrator.

Other:
